# Novel Vascular Malformation in an Affected Newborn with Deletion Del(4)(q31.3)

**DOI:** 10.1155/2012/321569

**Published:** 2012-12-25

**Authors:** Norma Elena de León Ojeda, Michel Soriano-Torres, Mercedes J. Cabrera, Dunia Bárbara Benítez Ramos

**Affiliations:** ^1^Hospital Pediátrico William Soler, Calle San Francisco Esquina Perla, Altahabana, Boyeros, La Habana, Cuba; ^2^Centro Nacional de Genética Médica, Avenida 31 y Calle 146, Cubanacán, Playa, La Habana, Cuba

## Abstract

We report on a newborn male patient with a terminal deletion in the long arm of the chromosome 4 with a congenital heart defect unreported before in association with this syndrome. The patient had multiple congenital anomalies including a pointed duplicated fingernail, low set posteriorly rotated ears, large anterior fontanel, micrognathia, glabellar capillary vascular malformation, and Interrupted Aortic Arch type C. The patient died due to multiple congenital malformations; a peripheral chromosome analysis showed 46, XY, del(4)(q31.3) *de novo*. The only reported case with the same deletion was a male newborn that exhibited the pattern of minor anomalies of deletion 4q31 syndrome. The parents were cytogenetically normal. We compare clinical signs to other cases with a deletion in long arm of chromosome 4.

## 1. Introduction

The 4q syndrome has been well characterized; its main manifestations are the Robin sequence, microcephaly, typical facial features, developmental delay, central nervous system anomalies, cryptorchidism, limb and genitourinary anomalies, short stature, fifth finger clinodactyly, and variable congenital heart defects [1, 2]. Some other features have been reported such as bilateral optic nerve hypoplasia in association with this syndrome [3]. Other defects associated to this syndrome have been described in 25 patients around the world with different breakpoints. The incidence of terminal deletion of chromosome 4q is believed to be very low but is not certainty known, only a limited number of reports describing such deletions exist [4].

During the neonatal period babies have severe respiratory troubles such as laryngeal hypotonia and oropharyngeal incoordination and up to 50% die before 15 months of age [5].

Although some authors have suggested that phenotype severity is directly proportional to the size of the deleted segment, it has been demonstrated that the 4q phenotype is not restricted to terminal deletions and severity is not necessarily related to the extent of the missing segment [6]. The features of this syndrome may not always be present and therefore misdiagnosis may occur without genetic consultation in subjects with 4q deletion syndrome who display minor phenotypic features, which could be the reason so few cases have been described thus far [7].

## 2. Case Presentation

**Figure 1:**
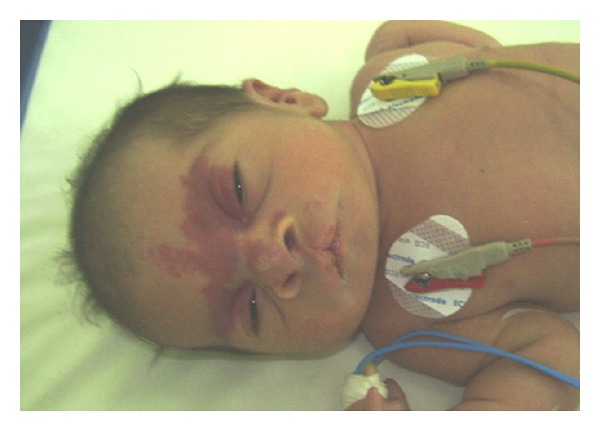
Phenotypical findings described on the text. (With parent's permission).

**Figure 2:**
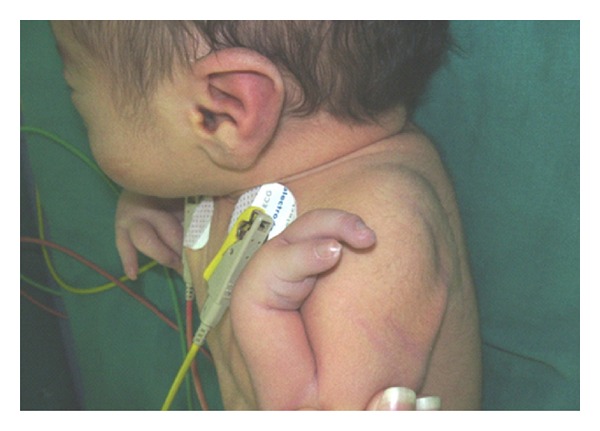
Right and left hands. Note the fusiform appearance of fingers and hyperconvex nail with a curvature towards hypoplastic pulps.

**Figure 3:**
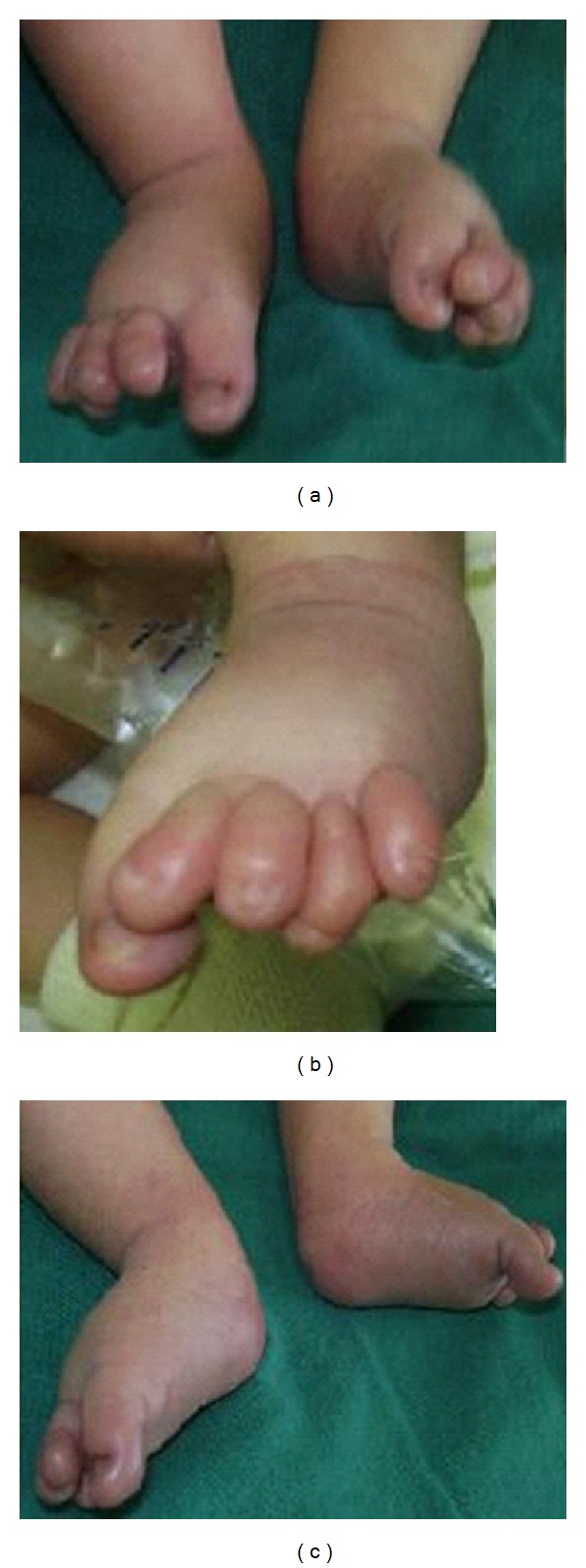
Right and left feet. Note swelling at dorsum with tibial clinodactyly of both second toes, hypoplastic nails, and tapering of second over hallux and third and fifth over fourth toes bilateral and symmetrically. Archives of Drs. de León NE and Benítez DB. William Soler cardiocentre. With the consent of the parents.

The patient was a 5-day newborn (Figure 1), first child of healthy, nonconsanguineous, and phenotypically normal parents with an unremarkable reproductive and family history. There was no prenatal history of medication, alcohol intake, or smoking. He was delivered at term, vaginally, and normal height and weight. At birth the baby had a cardiovascular event, requiring cardiologist evaluation and had also clinically depressed metopic suture, glabellar hemangiomatous malformation in oporto wine that extends toward the nose, metopic suture, and supraorbital ridges; the hair in the frontal area tends to be implanted as a widow's peak, ocular hypertelorism, broad and high nasal bridge, shallows orbits with hypoplastic supraorbital ridges and malar regions, wide base of the nose, anteversion narines, nonmidline cleft of the upper lip at left, without cleft of alveolar ridges, palate or uvula, pointed chin, and large appearance of ears. Limbs anomalies were longitudinal distal meromelia of left hand and forearm involves absent radii, metacarpal and phalanges from 3 to 5 fingers, a right radial hypoplasia, a left radial agenesis, lymphedema of the dorsum of feet, bilateral tapering of four over 5 and 3 toes (Figure 2), and right inguinal hernia. The fifth finger of the right hand had hyperconvex nail with a curvature toward a hypoplastic pulp and a fusiform appearance; the same sign is observed in the left hand but in thumb with a hyperconvex nail and hypoplastic pulp. Feet were swelling at dorsum with tibial clinodactyly of both second toes (Figure 3), hypoplastic nails, and tapering of second over hallux and third and fifth over fourth toes bilaterally and symmetrically. The patient had also Interrupted Aortic Arch (IAA) type C, a complex cardiac defect. A complete description of the signs observed in our case and compared with other defects previously described in association with this syndrome is presented in Table 1.

**Figure 4:**
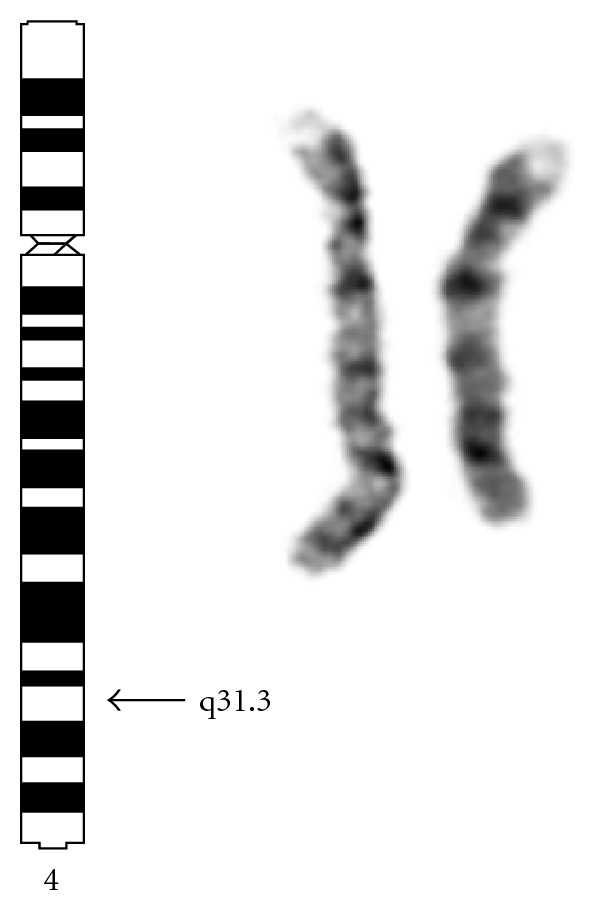
Partial karyotype of the patient, schematic presentation of chromosome 4, right: normal and deleted chromosomes 4.

Cytogenetic analysis was performed on cultured PHA-stimulated peripheral blood lymphocytes of the propositus and his parents according to standard techniques. The lymphocyte culture showed a good mitotic index and classical cytogenetics was carried out using GTG banding [8]. The karyotype was 46,XY,del (4)(q31.3) *de novo,* observed on a resolution of 400 bands. Both parental karyotypes were normal. Molecular analysis of the origin of the deleted 4q was not undertaken. A partial karyotype is presented in Figure 4.

## 3. Discussion

Because of hand findings the first suspected clinical diagnosis was a deletion of the long arm of chromosome 4. The fifth finger with either a hooked or a volar nail is a distinctive feature in distal 4q34 deletions [1]; when present, this single clinical feature should bring to mind the possibility of a terminal 4q deletion [9]. 

Most of previous reports of deletion of the long arm of chromosome 4 [9–11] have involved proximal regions (4q11–q31) and are generally associated to less clinical severity and lower mortality than are described in larger deletions. Robertson considers the critical region for the phenotype 4q syndrome to be 4q31.22–4q34.2 [6]. The only one case with the same breakpoints (4q31.3) of the patient presented was reported in a male newborn who exhibited the pattern of minor anomalies of the 4q deletion syndrome, but without associated major malformations [12].

Terminal deletion of chromosome regions 4q31, 4q32, and 4q33 has also been recognized by different authors as a distinctive malformation syndrome including variable mental and growth deficiency, cleft palate, limb anomalies, and cardiac and genitourinary defects [6, 9, 11]. 

Other complex intrachromosomal 4q rearrangement involving 4q34.1 deletion had a phenotype with retrognathia, congenital heart defect, ear fistula, and suggested gene/s relevant for the facial and cardiac phenotype could be mapped in the region 4q33 or proximal 4q34.1 [13].

Limb deficiencies are present in about 30–60% of the patients with 4q33 deletions and are associated with a gene tentatively assigned to this region [14]. The major limb anomalies are uni- and bilateral ulnar ray defects and minor anomalies including fifth digit clinodactyly of the hands and feet, hypoplastic nails, and overlapping toes [15]. At the present case the ray defects were bilateral but radial with left agenesis and right hypoplasia of radius. Ectrodactyly of hands as also lymphedema of the dorsum of feet and overlapping toes are previously reported in other cases [1, 4, 9, 11, 16].

Most common anomalies observed in these patients are craniofacial (99%), digital (88%), skeletal (54%), and cardiac (50%) [17]. Facial appearance is similar to cases reported previously [9, 16]. Facial features include a high forehead in 73% of index cases [18], although glabellar capillary hemangiomatous malformation was previously reported as glabellar hemangioma in two cases. *Vascular Endothelial Growth Factor C *(*VEGFC*) encodes a platelet-derived growth factor/vascular endothelial growth factor, which is active in angiogenesis and endothelial cell growth. Deletion of *VEGFC* may be associated with development of the glabellar hemangioma [19].

Cardiovascular malformations such as right ventricular outflow tract obstruction are present in 61% of these patients. It has been postulated that genes distal to 4q34 may play a critical role causing heart defects, although the molecular bases are not known. Deletion of dHAND, a transcription factor for ventricular cardiomyocyte expansion mapped to 4q33, might contribute to heart and limb defects, but not all the patients with dHAND deletions show these phenotypes [19, 20]. 

Some heart defects reported in other cases are ventricular septal defects, patent ductus arteriosus, peripheral pulmonic stenosis, aortic stenosis, tricuspid atresia, atrial septal defect, aortic coarctation, and tetralogy of Fallot. The complex cardiovascular defect with IAA type C described at the present case is reported by first time in association with 4q syndrome. There is no direct association of clinical findings with a target and different bands but dHAND gene plays an important role in the development of brachial arches and craniofacial structures [13]. *SORBS2* encodes a protein containing N-terminal sorbin and a C-terminal SH3 domain and is highly expressed in epithelia and cardiac muscle tissue. It suggests that this gene may play an important role in heart development and may potentially contribute to congenital heart disease in patients with 4q deletion syndrome [19]. Therefore, it has been proposed that IAA type C may be rare feature of this syndrome.

The parent's chromosomes are usually normal; few articles have described chromosomal rearrangements in parents and sons; it was present in two families and a mother with a pericentric inversion [9, 21]. The previous reports also indicate that there are more patients with large terminal deletions for full expression of the 4q phenotype, while there are less number of cases involving interstitial 4q31 deletions to be proposed as the “minimal critical region” most likely responsible for the 4q syndrome [6].

Although the breakpoints occurred at different levels of the chromosome, the clinical findings described for each type of breakpoint deletions were rather similar in that they comprised variable mental and growth retardations with phenotypical characteristics involving craniofacial, digital, skeletal, and cardiac anomalies. Therefore, it was difficult to determine clinically the level of breakpoint based on the clinical features. A precise genotype-phenotype correlation at this region has been hampered because most of the deletions reported until now, with few exceptions, have been characterized only at the cytogenetic level [9]. 

The present case, according with a terminal deletion in the breakpoint q31.3, had a severe phenotype that ended in a premature death at 5 days after birth. It has been described that surgery procedures for complications in this syndrome patients with the use of bilateral distraction osteogenesis of the mandible in order to increase posterior airway space allowed a patient to ventilate without any adjuncts or mechanical ventilation assistance [22] but it was not necessary in the patient.

## Figures and Tables

**Table 1:** Clinical signs in this patient previously described in the literature.

Clinical signs described in other patients	Clinical signs observed in our patient
Intrauterine growth restriction (83%)	
Mental retardation (92%)	
Hypotonia (28%)	**×**
Seizures (17%)	
Large anterior fontanel	**×**
Ocular hypertelorism (56%)	**×**
Short nose (67%)	**×**
Broad nasal bridge (94%)	
Cleft lip/palate (94%)	**×**
Micrognathia (94%)	**×**
Low set posteriorly rotated ears (56%)	**×**
Abnormal pinnae (67%)	
Fifth finger clinodactyly (44%)	**×**
Tapering fifth finger (50%)	
Pointed duplicated fifth fingernail (33%)	**×**
Absent to hypoplastic flexion creases of fifth finger	**×**
Abnormal thumb or hallux implantation (44%)	**×**
Simian crease (61%)	
Overlapping toes (22%)	**×**
Cardiac defects (61%)	**×**
Genitourinary defects (50%)	
Gastrointestinal defects (22%)	
Occasional:	
Asymmetric face (17%)	
Epicanthal folds (39%)	**×**
Anteverted nares (33%)	**×**
Cleft lip (39%)	**×**
Up slanting palpebral fissures (22%)	**×**
Missing digits (11%)	**×**
Robin sequence (17%)	
Pointed helix (faun-like ears appearance)	

## References

[1] Vogt J, Ryan E, Tischkowitz MD, Reardon W, Brueton LA (2006). The tale of a nail sign in chromosome 4q34 deletion syndrome. *Clinical Dysmorphology*.

[2] Strehle EM, Ahmed OA, Hameed M, Russell A (2001). The 4q-syndrome. *Genetic Counselling*.

[3] Parentin F, Fabretto A, Benussi DG (2009). Ophthalmic features in a dysmorphic boy with chromosome 4q deletion and duplication. *Ophthalmic Genetics*.

[4] Sills ES, Burns MJ, Parker LD (2007). Further phenotypic delineation of subtelomeric (terminal) 4q deletion with emphasis on intracranial and reproductive anatomy. *Orphanet Journal of Rare Diseases*.

[5] Jones KW (2006). *Smith's Recognizable Patterns of Human Malformation*.

[6] Robertson SP, O’Day K, Bankier A (1998). The 4q-syndrome: delineation of the minimal critical region to within band 4q31. *Clinical Genetics*.

[7] Caliebe A, Waltz S, Jenderny J (1997). Mild phenotypic manifestations of terminal deletion of the long arm of chromosome 4: clinical description of a new patient. *Clinical Genetics*.

[8] Verna RS, Babu E (1995). *Human Chromosomes. Principles and Techniques*.

[9] Lin AE, Garver KL, Diggans G (1988). Interstitial and terminal deletions of the long arm of chromosome 4: further delineation of phenotypes. *American Journal of Medical Genetics*.

[10] Eggermann K, Bergmann C, Heil I, Eggermann T, Zerres K, Schüler HM (2005). Rare proximal interstitial deletion of chromosome 4q, del(4)(q13.2q21.22): new case and comparison with the literature. *American Journal of Medical Genetics*.

[11] Kaalund SS, Møller RS, Tészás A (2008). Investigation of 4q-deletion in two unrelated patients using array CGH. *American Journal of Medical Genetics Part A*.

[12] Schinzel A (2001). *Catalogue of Umbalanced Chromosome Aberrations in Man*.

[13] Sensi A, Prontera P, Buldrini B (2008). Cytogenetic and array CGH characterization of an intrachromosomal complex rearrangement of 4q in a patient with a 4q-phenotype. *American Journal of Medical Genetics Part A*.

[14] Kocks A, Endele S, Heller R (2002). Partial deletion of 4p and 4q in a fetus with ring chromosome 4: phenotype and molecular mapping of the breakpoints. *Journal of Medical Genetics*.

[15] Mdzin R, Ko C, Abdul Latif Z, Zakaria Z (2008). Interstitial deletion of the distal long arm of chromosome 4, del (4)(q33–q35), in association with paternal balanced translocation. *Singapore Medical Journal*.

[16] Balcı S, Engiz O, Aktaş D (2006). Ring chromosome 4 and Wolf-Hirschhorn syndrome (WHS) in a child with multiple anomalies. *American Journal of Medical Genetics*.

[17] Strehle EM, Bantock HM (2003). The phenotype of patients with 4q-syndrome. *Genetic Counseling*.

[18] Strehle EM (2011). Dysmorphological and pharmacological studies in 4q- syndrome. *Genetic Counseling*.

[19] Strehle EM, Yu L, Rosenfeld JA (2012). Genotype-phenotype analysis of 4q deletion syndrome: proposal of a critical region. *American Journal of Medical Genetics*.

[20] Huang T, Lin AE, Cox GF (2002). Cardiac phenotypes in chromosome 4q- syndrome with and without a deletion of the *dHAND* gene. *Genetics in Medicine*.

[21] Stembalska A, Laczmanska I, Schlade-Bartusiak K, Czemarmazowicz H, Murawski M, Sasiadek M (2007). Recombinant chromosome 4 resulting from a maternal pericentric inversion in two sisters presenting consistent dysmorphic features. *European Journal of Pediatrics*.

[22] Markiewicz MR, Verschueren D, Assael LA (2010). Chromosome 4q deletion syndrome: craniofacial characteristics associated with monosomy of the long arm of chromosome 4q. *Cleft Palate-Craniofacial Journal*.

